# COVID-19 Pandemic Impact on Nursing Homes Financial Performance

**DOI:** 10.1177/00469580241240698

**Published:** 2024-03-21

**Authors:** Gregory N Orewa, Robert Weech-Maldonado, Justin Lord, Ganisher Davlyatov, David Becker, Sue S. Feldman

**Affiliations:** 1 University of Texas San Antonio, San Antonio, TX, USA; 2 University of Alabama at Birmingham, Birmingham, AL, USA; 3 Louisiana State University in Shreveport, Shreveport, LA, USA; 4 University of Oklahoma Health Sciences Center, Oklahoma City, OK, USA

**Keywords:** long-term care, nursing homes, COVID-19, financial performance, operating margin, operating revenue, operating cost

## Abstract

Nursing homes expressed concern about potential severe adverse financial outcomes of COVID-19, with worries extending to the possibility of some facilities facing closure. Maintaining a strong financial well-being is crucial, and there were concerns that the pandemic might have significantly impacted both expenses and income. This longitudinal study aimed to analyze the financial performance of nursing homes during COVID-19 pandemic. Specifically, we examined the impact of the pandemic on nursing home operating margins, operating revenue per resident day, and operating cost per resident day. The study utilized secondary data from various sources, including CMS Medicare cost reports, Brown University’s Long Term Care Focus (LTCFocus), CMS Payroll-Based Journal, CMS Care Compare, Area Health Resource File, Provider Relief Fund distribution data, and CDC’s NH COVID-19 public file. The sample consisted of 45 833 nursing home-year observations from 2018 to 2021. Fixed-effects regression analysis was employed to assess the impact of the pandemic on financial performance while controlling for various organizational and market characteristics. The study found that nursing homes’ financial performance deteriorated during the COVID-19 pandemic. Operating margins decreased by approximately 4.3%, while operating costs per resident day increased by $26.51, outweighing the increase in operating revenue per resident day by about $17. Occupancy rates, payer mix, and staffing intensity were found to impact financial performance. The study highlights the significant financial impact of the COVID-19 pandemic on nursing homes. While nursing homes faced substantial financial strains, the findings offered lessons for the future, underscoring the need for nursing homes to improve the accuracy of their cost reports and enhance financial transparency and accountability.

**What do we already know about this topic?**
The financial performance of nursing homes was significantly disrupted by the havoc wrought by COVID-19, though the exact extent of this impact remains uncertain.
**How does your research contribute to the field?**
The findings from our study enhance the general understanding of how nursing homes were affected in terms of the magnitude of changes in revenue, costs, and operating margins.
**What are your research’s implications toward theory, practice, or policy?**
Understanding the financial consequences of the COVID-19 pandemic on nursing homes is essential for developing a thorough and efficient strategy to address similar health crises in the future.

## Introduction

Nursing homes face heightened internal and external pressures such as staffing requirements, changing reimbursement methodology, declining occupancy, increasing competition, and ever-changing regulations, which have contributed to a challenging landscape for these organizations.^1-6^ As such, concerns about the financial viability of nursing homes have come to the forefront.^2^ A recent study by Hughes et al shows that from 2011 to 2021, there were approximately 1459 nursing home closures, or 8.9% of the study sample. Urban facilities and those with higher proportions of racial/ethnic minorities and Medicaid residents were at higher risk of closure.^7^ The heightened anxiety that surrounded COVID-19 sparked significant concerns about potential financial consequences for nursing homes with worries extending to the possibility of some facilities facing closure.^8-10^

COVID-19 may have affected both costs and revenues. On the cost side, demand for personal protective equipment (PPE) and cleaning supplies skyrocketed during the pandemic, and suppliers could not keep up with the demand.^11-13^ Due to disruptions in both supply and demand, nursing homes not only grappled with increased costs for essential supplies, but also competed with other healthcare organizations for critical medical instruments, surgical supplies, and PPE, leading to widespread shortages.^11-13^ During this crisis, nursing homes had no choice but to purchase supplies at inflated prices. Even when the federal government stepped in to intervene using the Defense Production Act to increase PPE and supplies, hospitals were the primary priority while nursing homes were secondary or even further down the priority line.^14^ Additionally, the existing staffing shortages in nursing homes, which were already a challenge before the pandemic, were intensified. To address the increased demand for care, facilities had to resort to hiring contract nurses through agencies, potentially incurring higher labor costs.^15-21^

On the revenue side, nursing homes may have had a decline in post-acute admissions during the pandemic since they were hesitant to accept new residents for fear of spreading the virus.^22,23^ Hospitals that tried to discharge patients quickly received pushback from nursing homes.^22,24,25^ The unwillingness to accept new and/or returning residents and the increased death rate may have resulted in decreased occupancy with potential impact on revenues.^24,26^ On the other hand, during the Public Health Emergency (PHE), the Centers for Medicare and Medicaid Services (CMS) introduced waivers that may have positively impacted Medicare reimbursement for nursing homes. One of these waivers eliminated the requirement for a 3-day hospitalization before a skilled nursing admission. This allowed patients to be admitted directly to a nursing home from the community or an emergency department to receive skilled nursing care.^27^

Furthermore, the intervention of the federal government through the Coronavirus Aid, Relief, and Economic Security (CARES) Act funding was essential in providing much-needed support to nursing homes during the pandemic. Even with its positive impact, the CARES Act funding may have been insufficient in fully addressing all the challenges faced by some facilities.^10,28,29^ The distribution of this funding was not entirely equitable, as it was primarily distributed through the Provider Relief Fund, using a formula based on total revenue and Medicare revenue to allocate funds to facilities.^29-31^ Regardless of efforts to distribute funds based on need, some nursing homes with smaller budgets or those serving primarily Medicaid patients may not have received enough funding to address the challenges posed by the pandemic. On the other hand, concerns were raised about the potential exaggeration of financial challenges by some facilities to secure more funds and the possible misuse of these funds.^4,29,32^

Despite the challenges posed by increased costs and low occupancy rates during the pandemic, an interesting trend of buying and selling nursing homes emerged, reflecting investors’ perception of these facilities as potentially lucrative assets.^33^ Notably, Ensign, the largest publicly traded nursing home entity, reported a significant increase in both stock prices and profit margins^33^ This trend was also observed among many Real Estate Investment Trusts (REITs) with ownership stakes in nursing homes, experiencing similarly elevated financial performance during this period.^33^

To date, there has been limited research examining the impact of COVID-19 on the financial performance of nursing homes. A study by Kingsley and Harrington of publicly traded nursing home companies from 2018 to 2020, showed that COVID-19 had little financial impact.^28^ In another study of California nursing homes between 2019 and 2020 showed that some nursing homes had a substantial increase in profit margins between 2019 and 2020.^34^ Using national, longitudinal data from 2018 to 2021, this study analyzed the impact of COVID-19 on nursing homes financial performance. Financial stability is critical for any successful organization. Sustained, poor financial performance puts an organization’s solvency in question.^2,35^ Monitoring the continual dynamics of nursing home finances is critical to understanding how nursing homes are faring given the ever-changing environmental climate in which they operate.

## Method

### Data Sources

This study utilized secondary data from 7 different sources: CMS Medicare Cost Reports, Brown University’s Long Term Care Focus (LTCFocus), CMS Payroll-Based Journal (PBJ), CMS Care Compare, Area Health Resource File (AHRF), Provider Relief Fund distribution from U.S. Department of Health and Human Services (HHS), and NH COVID-19 public file from Centers for Disease Control and Prevention (CDC) from 2018 to 2021. Medicare Cost Reports provides financial data for nursing homes that participate in the Medicare program. LTCFocus data provides nursing home organizational information such as occupancy rate, acuity index and racial/ethnic mix. CMS Care Compare contains facility information such as RN skill mix, and star ratings. The Payroll-Based Journal (PBJ) is a database of staffing records which is audited.^36^ Nursing homes are required to report total direct care hours spent as well as the facility census to the PBJ.^36^ The Area Health Resource File (AHRF) contains market and demographic data at the county level. Provider Relief Fund HHS provides data on government financial assistance given to each nursing home during COVID-19. The COVID-19 public file contains Total Resident Confirmed COVID-19 Cases per 1000 Residents reported on nursing homes by CDC.

### Study Sample

The study sample included all Medicare and Medicaid certified SNFs from 2018 to 2021, or 62 331 nursing home-year observations. Hospital-based nursing homes and federal government nursing homes (n = 2311) were excluded because these institutions may have different organizational structures and operating environment than free-standing facilities. Additionally, their nursing home cost reports are part of overall hospital cost reports and are not included in the Medicare nursing home cost reports. After merging all datasets, the final analytical sample was 45 833 nursing home-year observations or an average of 11 458 nursing homes per year. The study period comprised the pre-pandemic period of 2018 to 2019 and the pandemic period of 2020 to 2021.

### Measures

Table 1 provides a comprehensive overview of the operationalization and data sources of all the variables used in the study. The dependent variables in this study are operating revenue per resident day, operating cost per resident day, and operating margin. The operating revenue per resident day was calculated by dividing the operating revenue by the total inpatient days. The operating cost was calculated by first deducting the Medicare disallowed expenses—which are specific cost or charges that are not permitted under Medicare reimbursement guidelines^37,38^—and then dividing the adjusted operating expense by the total inpatient days. Operating margin was calculated by subtracting operating revenue from operating expense and then dividing it by operating revenue. To address outliers in the financial data, we examined the operating revenues and operating costs and removed observations that were 5 standard deviations or more from the mean.

**Table 1.** Variables Operationalization and Data Sources.

Variable names	Operationalization	Data source
*Dependent variable*		
Operating revenue per resident day	Operating Revenue/Total Inpatient Day	CMS Cost Report
Operating cost per resident day	Operating Cost/Total Inpatient Day	CMS Cost Report
Operating margins	(Operating Revenue – Operating Cost)/Operating Revenue	CMS Cost Report
*Independent variable*		
COVID-19 pandemic	Pre-COVID-19 and COVID-19 years	Pre-COVID-19 – 2018-2019 COVID-19 – 2020-2021
*Organizational-level control variables*		
Occupancy rate	Percentage of bed occupied in the nursing home	LTC Focus
Payer mix	Primary Payer of the Proportion of inpatient day: Medicare, Medicaid, or Private Pay.	CMS Cost Report
Acuity index	Measure of the average level of care needed by resident	LTC Focus
Racial/ethnic mix	Percentage of nursing home residents who are White, Black, Hispanic, and other race/ethnicity	LTC Focus
Staffing intensity	Reported RNs, LPNs, and CNAs, hours per 100 resident per day	Payroll Based Journal
RN skill mix	Proportion of RNs to total nursing staff	CMS Care Compare
Quality of care	Quality measure five-star rating	CMS Care Compare
*Market-level control variables*		
Herfindahl-Hirschman Index	The total number of beds divided by the sum of all the county beds squared, and then this sum is divided by the sum of all county bed squared	Area Resource File
Medicare Advantage (MA) penetration rate	Total number of MA plan enrollees divided by total number of Medicare beneficiaries	Area Resource File
Per capita income ($)	Residents’ total income divided by the population of the area	Area Resource File
Percent of individual 65+	The percent of individuals who are 65 and older is the proportion of all residents 65 and older to the total nursing home population	Area Resource File
*COVID-19 related variables*		
CARES act relief fund	COVID-19 related payment to SNF	Provider Relief Fund HHS
COVID-19 SNF cases	Nursing homes reported confirmed COVID-19 cases per 1000 residents	Nursing Home COVID-19 Public file

The independent variable consisted of the COVID-19 pandemic, and it represents the 2-year period of the pandemic 2020 to 2021. The reference group, pre-pandemic represents the 2-year period before COVID-19 (2018-2019). Given that the pandemic may have had a disproportionate effect on facilities with greater reliance on post-acute care, we included an interaction between COVID-19 pandemic variable and high Medicare. High Medicare is a dichotomous variable, where 1 = 25% or higher Medicare census “top 25th percentile” and 0 = less than 25% Medicare census “bottom 75th percentile.”

Control variables widely used in nursing home research literature were included in the model based on factors that could impact financial performance.^39-44^ Organizational control variables included occupancy, payer mix, acuity index, resident racial/ethnic mix, nurse staffing, and quality measures star rating. Market control variables included the Herfindahl-Hirschman index, Medicare Advantage (MA) penetration rate, per capita income, percent of individuals 65 and up. In addition, the study controlled for COVID-19 factors that may have affected revenues and costs, such as the CARES Act relief funding and COVID-19 cases at the nursing home facility level.^45-47^

The occupancy rate was computed by dividing the number of occupied beds by the total number of beds in the nursing home. Payer mix is the percent of residents who are Medicaid. The Acuity Index is a measure of the level of care required by nursing home residents; it is determined by the number of residents who require various levels of assistance, such as bed mobility, shifting from bed to chair, eating/toileting, and those who require specific treatment, such as physical therapy or tube feeding. Proportion of racial/ethnic minorities is the proportion of nursing home residents who were White, Black, Hispanic, and other race/ethnicity. Nurse staffing reflects: RN hours per resident day (PRD), licensed practical nurse (LPN) hours PRD and certified nursing assistant (CNA) hours PRD. RN skill mix was measured as the proportion of RNs hours to the total nursing staffing hours. The quality measures star rating was obtained from the CMS Care Compare Five-Star Quality Rating System. Ratings for the quality measures are based on performance on 15 quality measures: 9 long-stay measures and 6 short-stay measures. The Care Compare website assigns a score to nursing homes ranging from 1 (lowest quality) to 5 (highest quality). Nursing homes with a star rating of 1, are below average, while nursing homes with a star rating of 5, are above average.

The Herfindahl-Hirschman index (HHI) is a continuous variable that ranges from 0 to 1. HHI is calculated as the sum of the market shares of nursing homes in a county. When the index is closer to 1, it signifies a less competitive market.^44^ Medicare Advantage (MA)/managed care market penetration is measured as the percentage of all Medicare beneficiaries in the county enrolled in an MA plan. Per capita income is measured by an individual’s average wealth in the county. The percent of individuals who are 65 years and older is the proportion of all residents 65 years and older to the total nursing home population.

The COVID-19 control factor variables used in this study are available only in the post-period such as the COVID-19 relief funding to nursing home by the federal government during the pandemic, and the total resident confirmed COVID-19 cases per 1000 residents at the nursing home facility level.

### Analysis

We focused on nursing homes as our unit of analysis. Descriptive statistics and univariate analysis were performed for each of the variables. Continuous variables were evaluated using mean and standard deviation, while categorical variables were evaluated using frequency and percentages. Bivariate analysis was used to compare the study variables in the pre-COVID-19 and COVID-19 period. The data were modeled using fixed-effects regression to account for the time invariant and unobservable variables. The study controlled for time trends using year fixed effects, and robust clusters were used to address correlation within groups at the facility level.^48^ We also adjusted for inflation using the CPI inflation index. Stata v17 was used to conduct the analysis of the study. *P*-value < .05 was considered statistically significant.

## Results

Table 2 shows the bivariate statistics for the study sample. Operating revenue per resident day increased from $317 pre-COVID-19 to $328 during COVID-19. Operating cost per resident day increased from $308 pre-COVID-19 to $343 during COVID-19. Nursing homes operating margins decreased from about 2.7% pre-COVID-19 to −3.0% during COVID-19. There was a decline in occupancy rate from 81% pre-COVID-19 to 74% during this COVID-19. The proportion of nursing homes that had percent Medicare less than 25 and greater than 25 did not experience significant change. Medicaid increased from 55.7% pre-COVID-19 to 58.3% during COVID-19, and those covered by other financing sources declined from 31% pre-COVID-19 to 29.6% during COVID-19. The average case-mix (Acuity Index) for the nursing home population remained constant. Nursing home residents that were White decreased from 77.9% pre-COVID-19 to 76.3% during COVID-19, while Black (10%), Hispanic (2.9%), and other race (9%) pre-COVID-19 increased only slightly during COVID-19.

**Table 2.** Bivariate Statistics of Study Sample (N = 45 833) Nursing Homes Observations.

Variables	Pre-COVID-19 (2018-2019) N = 25 027	COVID-19 (2020-2021) N = 20 806	*P*-value
*Dependent variable*			
Operating revenue per resident day	317.32 (172.99)	327.90 (157.84)	<.001
Operating cost per resident day	308.06 (174.57)	343.11 (197.79)	<.001
Operating margin	2.72 (12.16)	−3.05 (17.54)	<.001
*Organizational-level control variable*			
Occupancy rate	80.58 (14.25)	73.50 (16.30)	<.001
Payer mix			
Medicaid	55.68 (26.30)	58.35 (24.36)	<.001
Medicare			
<25% Medicare	11 475 (90.78)	8007 (90.94)	<.001
>25% Medicare	1166 (9.22)	798 (9.06)	<.001
Private	31.15 (23.25)	29.60 (22.19)	<.001
Acuity Index	12.23 (1.25)	12.15 (1.94)	<.001
Racial/ethnic mix			
Whites	77.87 (24.47)	76.34 (24.47)	<.001
Black	10.13 (19.06)	10.89 (19.52)	<.001
Hispanic	2.95 (10.50)	3.02 (10.42)	.464
Other race	9.04 (13.17)	9.75 (13.58)	<.001
RN hours per resident day	0.41 (0.28)	0.44 (0.29)	<.001
LPN hours per resident day	0.79 (0.29)	0.81 (0.30)	<.001
CNA hours per resident day	2.16 (0.49)	2.02 (0.54)	<.001
RN skill mix	11.78 (6.87)	13.20 (7.30)	<.001
Quality measure star rating			
*	1107 (8.73)	704 (5.44)	<.001
**	2246 (17.72)	1669 (13.13)	<.001
***	2934 (23.15)	2746 (21.22)	<.001
****	3029 (23.90)	3434 (26.54)	<.001
*****	3358 (26.50)	4355 (33.66)	<.001
*Market-level control variable*			
Herfindahl-Hirschman Index	0.23 (0.27)	0.26 (0.29)	<.001
Medicare Advantage (MA) penetration rate	33.62 (14.36)	38.18 (13.82)	<.001
Per capita income ($)	51 210 (15 268)	53 054 (16 025)	<.001
Percent of individuals 65+	17.26 (4.04)	17.68 (4.10)	<.001
COVID-19 related control variable			
CARES act relief fund	0	66 028 (102 778)	N/A
Total resident confirmed COVID-19 cases per 1000 residents	0	710.79 (1062.25)	N/A

Nursing staffing hours per resident day (HRD) for RN HRD increased during COVID-19 from 0.41 to 0.44 and LPN HRD increased from 0.79 to 0.81. However, for CNA there was a decrease from 2.16 to 2.02 during the same period. RN skill mix increased from 11.8% pre-COVID-19 to 13.2% during COVID-19. The number of nursing homes that had quality measure star rating ranging from 1 to 3 decreased during COVID-19, while the number of facilities with rating 4 to 5 increased during COVID-19. HHI of 0.2 remained relatively constant signifying highly competitive markets. The proportion of Medicare eligible patients enrolled in Medicare Advantage increased from 33.6% pre-COVID-19 to 38.2% during COVID-19. Per capita income increased from $51 210 per year pre-COVID-19 to $53 054 during COVID-19. The proportion of people aged 65 and up increased from 17.3% pre-COVID-19 to 17.7% during COVID-19. On average, nursing homes received about $66 028 in COVID-19 relief funds, and on average, there were about 711 total resident confirmed COVID-19 cases per 1000 residents in the nursing homes.

Table 3 presents the fixed effects regression results for nursing home operating revenue per resident day, operating cost per resident day and operating margin. Our results suggest that nursing financial performance deteriorated during COVID-19. On average, the operating margin was about 4.3% lower during the pandemic *(P* <* .001).* The impact of COVID-19 was associated with an increase in both operating cost per resident day and operating revenue per resident day. While operating revenue per resident day increased by $9.44 *(P* <* .001)*, operating costs per resident day also increased by $26.51 *(P* <* .001)* during COVID-19. This suggests that even though there was an increase in operating revenue per day, operating costs were also on the rise. A sensitivity analysis of the fixed effects model was conducted by excluding the control variables. The results show a very similar pattern to those obtained with the full model. Operating revenue per resident day increased by $15.80, operating cost per resident day increased by $31.49, and operating margin was 4.9% lower during the pandemic.

**Table 3.** Fixed Effect Regression (N = 45 833) Nursing Homes-Year Observations.

Variables	Operating revenue per resident day	Operating cost per resident day	Operating margin
*Dependent variable*			
Pre-COVID-19	Ref	Ref	Ref
COVID-19	9.444***	26.505***	−4.32***
*Organizational-level control variable*			
Occupancy rate	−0.214***	−1.038***	0.255***
Payer mix			
Private	Ref	Ref	Ref
Medicaid	−0.716***	−0.393***	−0.023***
Medicare			
<25% Medicare	Ref	Ref	Ref
>25% Medicare	93.053***	78.206***	4.531***
Acuity Index	0.715	0.437	0.022
Racial/ethnic mix			
Whites	Ref	Ref	Ref
Black	0.195	0.202	−0.010
Hispanic	0.450**	0.238	0.032
Other race	0.147*	0.083	0.006
RN hours per 100 resident day	0.241**	0.753***	−0.078***
LPN hours per 100 resident day	0.167***	0.482***	−0.100***
CNA hours per 100 resident day	0.041	0.137***	−0.046***
RN skill mix	−0.288	−0.318	−0.126*
Quality measure star rating			
*	Ref	Ref	Ref
**	1.980	0.340	0.581*
***	3.683	1.819	1.166***
****	3.321	−1.538	1.737***
*****	3.754	−2.479	2.049***
*Market-level control variable*			
Herfindahl-Hirschman Index	9.675	27.289***	0.830
Medicare Advantage (MA) penetration rate	−0.310	−0.259	−0.032
Per capita income (per $1000)	0.563	0.544	−0.022
Percent of individual 65+	1.587	2.307	0.044
COVID-19 related control variable			
CARES act relief fund (per $10 000)	−0.002	−0.001	0.005
COVID-19 SNF cases	0.001	−0.001	0.000

* *P* < .05. **P < .01. ***P < .001.

Table 4 shows the fixed effects regression results for the interaction of COVID-19 pandemic and high Medicare. The interaction was significant indicating that the effect of the pandemic varied based on the facilities’ degree of post-acute specialization. High Medicare facilities experienced an increase in operating revenue of $1.4 compared to $10.27 for those with low Medicare (Appendix 1). Cost increases for both groups were very similar at approximately $27. Finally, high Medicare facilities experienced a lower decline in operating margin (−2.5%) compared to low Medicare ones (−4.5%).

**Table 4.** Fixed Effect Regression with Medicare Interaction (N = 45 833) Nursing Homes-Year Observations.

Variables	Operating revenue per resident day	Operating cost per resident day	Operating margin
*Dependent variable*			
Pre-COVID-19	Ref	Ref	Ref
COVID-19	10.264***	26.480***	−4.510***
Medicare	97.920***	78.062***	3.403***
*Interaction variable*			
COVID-19*Medicare_Interaction	−8.891***	0.263	2.061***
*Organizational-level control variable*			
Occupancy rate	−0.215***	−1.038***	0.255***
Payer mix			
Private	Ref	Ref	Ref
Medicaid	−0.716***	−0.393***	−0.023***
Acuity Index	0.731	0.437	0.018
Racial/ethnic mix			
Whites	Ref	Ref	Ref
Black	0.197	0.202	−0.011
Hispanic	0.463**	0.238	0.029
Other race	0.157*	0.083	0.004
RN hours per 100 resident day	0.255**	0.752***	−0.081***
LPN hours per 100 resident day	0.167***	0.482***	−0.100***
CNA hours per 100 resident day	0.040	0.137***	−0.045***
RN skill mix	−0.338	−0.316	−0.115*
Quality measure star rating			
*	Ref	Ref	Ref
**	1.942	0.341	0.59*
***	3.595	1.822	1.186***
****	3.265	−1.536	1.75***
*****	3.611	−2.475	2.082***
*Market-level control variable*			
Herfindahl-Hirschman Index	9.736	27.288***	0.816
Medicare Advantage (MA) penetration rate	−0.331	−0.259	−0.027
Per capita income (per $1000)	0.607	0.543	−0.032
Percent of individual 65+	1.375	2.313	0.005
COVID-19 related control variable			
CARES act relief fund (per $10 000)	−0.015	−0.001	0.008
COVID-19 SNF cases	0.000	−0.001	0.000

* *P* < .05. **P < .01. ***P < .001.

In terms of the control variables, nursing homes with higher occupancy rates had both lower operating revenue and lower cost per resident day, as well as higher operating margin *(P* <* .001)*. Nursing homes with a higher percentage of Medicaid payers had a lower operating revenue per resident day, lower operating costs per resident day, and a lower operating margin *(P* <* .001).* Nursing home with greater nursing staffing intensity had both higher operating revenue and higher costs, as well as lower operating margin *(P* <* .001).* Nursing home with higher quality measure star rating had a higher operating margin *(P* <* .001)* compared to those which lower quality measure star rating.

## Discussion

The primary goal of this study was to assess nursing home financial performance during the COVID-19 pandemic. The longitudinal study looked at the financial performance of nursing homes from 2018 to 2021. We classified pre-COVID-19 as the years (2018-2019) and COVID-19 (2020-2021). This study’s findings will contribute to the body of knowledge, providing more insights into how nursing homes fared financially during the pandemic, as well as stress the importance of monitoring and paying greater attention to nursing home transparency and accountability of their finances.

The impact of COVID-19 on nursing homes’ operating costs per resident amounted to around $26.51. The increase in the cost to care for a nursing home resident may be partly attributed to unforeseen rises in demand for PPE, stemming from both price hikes and shortages.^14,47^ Despite the federal government intervention, nursing homes struggled to gain adequate access to supplies as hospitals were the top priority.^14^ Nursing homes, on the other hand, had to safeguard staff and residents, by purchasing more PPE than usual, resulting in increased operating costs.^47,49^ Several nursing homes were also penalized for not having appropriate PPE or if staff members became infected with COVID-19.^50^ In addition to the exacerbated staffing shortages that nursing homes already faced before the pandemic, they had to fulfill the increased demand for care caused by the pandemic, hiring more staff directly or through agencies and pay higher wages and agency fees,^15-21^ increasing the cost of providing care to residents.^24,25^

While our study revealed an increase in operating revenue per resident day of $9.44, this increase should be viewed with caution given that operating costs per resident day increased almost 3 times more than operating revenue per resident day. The occupancy rate decrease may have been a contributing factor, with our findings indicating a drop in occupancy rate of approximately 7%. This aligns with other research, which has also noted a reduction in nursing home occupancy rates during this period.^24,26^ Many families were hesitant to send their loved ones to nursing homes for fear of exposing them to the virus. Furthermore, some nursing homes may have refused admission from hospitals because they lacked the capacity to care for such residents, while other nursing homes may have specialized in caring for COVID-19 residents.

Our study also showed that nursing homes had negative operating margin during COVID-19. This can be attributed to the increased operational costs faced by nursing homes during the pandemic. The decline in the occupancy rate further exacerbated the situation, as lower occupancy rates directly impact revenues generated by nursing homes, ultimately leading to a reduced operating margin. Nursing homes had to navigate staffing challenges during the pandemic, marked by increased demand for healthcare workers and staff shortages due to illness or quarantine measures.^19^ These challenges resulted in higher labor costs, with facilities having to resort to paying overtime or hiring contract staff at premium rates.^21^ Although some nursing homes received financial assistance and relief through government programs, these measures may not have fully compensated for the extensive financial challenges posed by the pandemic. The combination of heightened operational costs, reduced occupancy, and increased staffing expenses may have contributed to the negative operating margin observed in nursing homes during the challenging period of COVID-19.

Our results also suggest that nursing homes were differentially affected by COVID-19 based on their Medicare payer mix. Nursing homes with a low percentage of Medicare residents (less than 25%) experienced a more substantial increase in operating revenue per resident day during COVID-19 than those with a high percentage of Medicare residents (25% and higher). The higher revenue per resident day among lower Medicare facilities may have been due to higher Medicaid rates in many states during the pandemic.^29,51^ However, the change in operating cost per resident day was similar for both types of facilities. While both high and low Medicare nursing homes experienced a decline in operating margin during COVID-19, the decline for low Medicare facilities was worse. This suggests that low Medicare nursing homes may have been disproportionately affect by the lower occupancy rate during the pandemic, resulting in a worse operating margin.

Nursing homes with higher occupancy had a higher operating margin. Having higher levels of occupancy could be a key strategic decision for nursing homes’ financial performance. Moreover, our findings suggest that nursing homes that can effectively manage their resources and implement cost-saving measures may have a better chance of achieving a higher operating margin.

Based on our study findings, nursing homes that had higher nursing staffing intensity had a lower operating margin, likely due to the increased labor costs associated with hiring staff or increasing staffing hours. While having more nursing staff may likely improve resident care and satisfaction,^19,52^ our results suggest that it may not necessarily lead to a higher operating margin. Research also shows that some nursing homes turned to contract nurses during COVID-19, with 78% of facilities reporting the use of agency nurse staff paying both agency fees and higher rates per hour for nursing staff.^19-21^

Our result also showed that nursing homes with higher quality measure star ratings were associated with higher operating margins. A plausible explanation for this association is that facilities with superior quality measures, as reflected in their quality measure star ratings, demonstrate effective management of residents, thereby promoting better overall health. Previous research has underscored the interconnectedness between the quality of care provided by nursing homes and their financial performance.^44^ In essence, the ability to maintain higher standards of care is not only beneficial to the health and well-being of residents but may also play a pivotal role in shaping the economic sustainability of these facilities.

### Managerial/Policy Implication

There are some managerial and policy implications from this study. First, our research shows that COVID-19 negatively impacted nursing homes’ financial performance by disrupting operations and finances. This highlights the impact of external environmental factors, such as the pandemic, on organizations. Second, even with federal government intervention during the pandemic, some nursing homes continued to experience financial hardship, demonstrating their vulnerability within our healthcare system. Nursing homes require special attention from policymakers during a crisis or another epidemic because they care for our most vulnerable people. Given the pandemic’s significant inflationary impact on the economy, policymakers must prioritize nursing home reimbursements. Reviewing Medicare and Medicaid reimbursement rates is critical now so that nursing homes can not only have the necessary funds to provide adequate care to their residents, but also attract and retain the requisite level of personnel.

Furthermore, considering the ongoing inflation and increasing labor expenses, it is crucial to closely monitor nursing home finances. The financial challenges faced by some facilities have the potential to lead to closures, which could have far-reaching implications, significantly impacting not only the nursing homes themselves but also posing broader consequences for the residents and the community. Finally, there is a need for nursing homes to enhance the accuracy of their cost reports and improve financial transparency and accountability. This necessity arises from the potential incentive for nursing homes to hide profits in order to secure potentially higher reimbursements from Medicare and Medicaid. In the absence of audits, nursing homes may manipulate their financial reports, involving practices such as under-reporting disallowances, concealing profits within related party costs, transferring assets to REITs and related parties, and portraying losses to minimize tax obligations.

### Limitations

This study has several limitations. First, shortly before the pandemic, CMS modified the way it paid for SNF services to encourage more value in SNF spending by implementing the Patient Driven Payment Model (PDPM). The impact of PDPM policy change may not be reflected in our data as we only tried to isolate the impact of COVID-19 on nursing home financial performance. Second, although we tried to consider COVID-19 related factors, there could be other variables related to COVID-19 that may have affected our study, which may be unknown to us and therefore where not considered. Third, there is a growing body of literature on nursing homes hiding profits in the related party transactions.^38^ Exploring the impact of related party transactions on financial performance was beyond the scope of this paper. However, further research is needed exploring this area. Fourth, though we controlled for state variation in our model, if there were any policy changes in the state during the period, it may not have been accounted for in the model. Lastly, since we relied on CMS Cost Report data sets for our analysis, there might have been timing differences in some of the reporting since the data is reported on a fiscal year.

## Conclusion

This study showed that nursing homes financial performance deteriorated during COVID-19 pandemic. COVID-19 further exposed some of the long-standing shortcomings in nursing homes, revealing severe flaws in how we care for our country’s most vulnerable population. On a positive note, there are many valuable lessons for nursing homes to learn from the COVID-19 pandemic. The pandemic has highlighted the need for nursing homes to improve the accuracy of their cost reports and enhance financial transparency and accountability. If nursing homes provide more transparency and show more accountability for how government money is spent, the government might be able to target resources where they are most needed during a pandemic.

## Supplemental Material

sj-docx-1-inq-10.1177_00469580241240698 – Supplemental material for COVID-19 Pandemic Impact on Nursing Homes Financial Performance Supplemental material, sj-docx-1-inq-10.1177_00469580241240698 for COVID-19 Pandemic Impact on Nursing Homes Financial Performance by Gregory N Orewa, Robert Weech-Maldonado, Justin Lord, Ganisher Davlyatov, David Becker and Sue S. Feldman in INQUIRY: The Journal of Health Care Organization, Provision, and Financing

## References

[1] Tyler DA Fennell ML. Rebalance without the balance: a research note on the availability of community-based services in areas where nursing homes have closed. Res Aging. 2017;39(5):597-611. doi:10.1177/0164027515622244 26685182 PMC4912472

[bibr2-00469580241240698] Lord J Landry A Savage GT Weech-Maldonado R. Predicting nursing home financial distress using the Altman Z-score. Inquiry: The Journal of Health Care Organization, Provision and Financing. 2020;57:1-9. 10.1177/0046958020934946 PMC7333488 32613878

[bibr3-00469580241240698] Feng Z Fennell ML Tyler DA Clark M Mor V. The care span: growth of racial and ethnic minorities in US nursing homes driven by demographics and possible disparities in options. Health Aff. 2011;30(7):1358-1365. doi:10.1377/hlthaff.2011.0126 PMC3785292 21734211

[bibr4-00469580241240698] Cenziper D Jacobs J Mulcahy S. Hundreds of nursing homes with cases of coronavirus have violated federal infection-control rules in recent years. The Washington Post. 2020.

[bibr5-00469580241240698] Castle NG Engberg J Lave J Fisher A. Factors associated with increasing nursing home closures. Health Serv Res. 2009;44(3):1088-1109. 19674434 10.1111/j.1475-6773.2009.00954.x PMC2699923

[6] Centers for Medicare & Medicaid Services (CMS), HHS. Medicare program; prospective payment system and consolidated billing for Skilled Nursing Facilities (SNF) final rule for FY 2019, SNF value-based purchasing program, and SNF quality reporting program. Final rule. Fed Regist. 2018;83(153):39162-39290. 30091551

[7] Hughes K Feng Z Li Q , et al. Rates of nursing home closures were relatively stable over the past decade, but warrant continuous monitoring. Heal Aff Sch. 2023;1(2):1-7. 10.1093/haschl/qxad025 PMC10986232 38756237

[8] Mathews AW Kamp J . Covid-19 pandemic hits nursing-home finances. Wall Street Journal. 2020. Accessed July 15, 2022. https://www.wsj.com/articles/covid-19-pandemic-hits-nursing-home-finances-11590604049

[bibr9-00469580241240698] Stulick A. Nursing home closures hit smaller markets as financial, staffing crisis deepens across industry. 2022. Accessed June 11, 2022. https://skillednursingnews.com/2022/06/nursing-home-closures-hit-smaller-markets-as-financial-staffing-crisis-deepens-across-industry/#:~:text=Skilled%20nursing%20facilities%20situated%20in%20small%20markets%20across,reform%20on%20the%20horizon%20with%20no%20monetary%20support.

[10] AHCA. Long Term Care Faces Worst Financial Crisis in Years; Closures Loom Without Additional Funding. American Health Care Association; 2021. Accessed July 15, 2022. https://www.ahcancal.org/News-and-Communications/Press-Releases/Pages/Long-Term-Care-Faces-Worst-Financial-Crisis-In-Years;-Closures-Loom-Without-Additional-Funding.aspx

[11] Ahmed J Malik F Bin Arif T , et al. Availability of personal protective equipment (PPE) among US and Pakistani doctors in COVID-19 pandemic. Cureus. 2020;12(6):1-14 10.7759/cureus.8550 PMC7357309 32670687

[bibr12-00469580241240698] Iyengar KP Vaishya R Bahl S Vaish A. Impact of the coronavirus pandemic on the supply chain in healthcare. Br J Healthc Manag. 2020;26(6):1-4.

[13] Zhu G Chou MC Tsai CW. Lessons learned from the COVID-19 pandemic exposing the shortcomings of current supply chain operations: a long-term prescriptive offering. Sustainability. 2020;12(14):5858.

[14] Abbasi J. Abandoned” nursing homes continue to face critical supply and staff shortages as COVID-19 toll has mounted. JAMA. 2020;324(2):123-125. doi:10.1001/jama.2020.10419 32525535

[15] Denny-Brown N Stone D Hays B Gallagher D. COVID-19 Intensifies Nursing Home Workforce Challenges. 2020.US Department of Health and Human Services Assistant Secretary for Planning and Evaluation Behavioral Health, Disability, and Aging Policy.

[bibr16-00469580241240698] Shen K McGarry BE Grabowski DC Gruber J Gandhi AD. Staffing patterns in US nursing homes during COVID-19 outbreaks. JAMA Heal Forum. 2022;3(7):e222151. doi:10.1001/jamahealthforum.2022.2151 PMC9308062 35977215

[bibr17-00469580241240698] Reinhardt JP Franzosa E Mak W Burack O. In their own words: the challenges experienced by certified nursing assistants and administrators during the COVID-19 pandemic. J Appl Gerontol. 2022;41(6):1539-1546. 35343299 10.1177/07334648221081124 PMC8958287

[bibr18-00469580241240698] Lien CY. Temp nurses go digital: Examining gig care in US nursing homes. Sociol Health Illn. 2023;45:542-559. 36575601 10.1111/1467-9566.13600

[bibr19-00469580241240698] Brazier JF Geng F Meehan A , et al. Examination of staffing shortages at US nursing homes during the COVID-19 pandemic. JAMA Netw Open. 2023;6(7):e2325993. 37498600 10.1001/jamanetworkopen.2023.25993 PMC10375301

[bibr20-00469580241240698] Heiks C Sabine N. Long term care and skilled nursing facilities. Del J Public Heal. 2022;8(5):144-149. 10.32481/djph.2022.12.032 PMC9894029 36751604

[21] Stulick A . Nursing homes use of staffing agencies soars during pandemic as workforce crisis deepens. Skilled Nursing News. https://skillednursingnews.com/2021/06/nursing-homes-use-of-staffing-agencies-soars-during-pandemic-as-workforce-crisis-deepens/

[22] Levine S Bonner A Perry A Melady D Unroe KT. COVID-19 in Older Adults: Transfers Between Nursing Homes and Hospitals. Journal of Geriatric Emergency Medicine. 2020.

[23] Gadbois EA Brazier JF Meehan A Grabowski DC Shield RR. “I don’t know how many nursing homes will survive 2021”: Financial sustainability during COVID-19. J Am Geriatr Soc. 2021;69(10):2785-2788. doi:10.1111/jgs.17380 34287843 PMC8447370

[24] Ouslander JG Grabowski DC. COVID-19 in nursing homes: calming the perfect storm. J Am Geriatr Soc. 2020;68(10):2153-2162. 32735036 10.1111/jgs.16784

[25] Grabowski DC Mor V. Nursing home care in crisis in the wake of COVID-19. JAMA. 2020;324(1):23-24. doi:10.1001/jama.2020.8524 32442303

[26] Paulin E. Nursing homes are filled with empty beds, raising more concerns about care. AARP, October 2021.

[27] Ulyte A Waken RJ Epstein AM , et al. Medicare skilled nursing facility use and spending before and after introduction of the public health emergency waiver during the COVID-19 pandemic. JAMA Intern Med. 2023;183(7):637-645. doi:10.1001/jamainternmed.2023.0770 37093607 PMC10126940

[28] Kingsley DE Harrington C. COVID-19 had little financial impact on publicly traded nursing home companies. J Am Geriatr Soc. 2021;69(8):2099-2102. doi:10.1111/jgs.17288 34028003 PMC8242457

[29] CMA. Nursing Facilities Have Received Billions of Dollars in Direct Financial and Non-Financial Support During Coronavirus Pandemic. Center for Medicare Advocacy; 2021. https://medicareadvocacy.org/report-snf-financial-support-during-covid/#_edn69

[bibr30-00469580241240698] Ochieng N Biniek JF Musumeci M Neuman T. Funding for health care providers during the pandemic: an update. 2022. https://www.kff.org/policy-watch/funding-for-health-care-providers-duringthe-pandemic-an-update

[31] HRSA. Past Targeted Distributions. Health Resources & Services Administration. https://www.hrsa.gov/provider-relief/payments-and-data/past-payments/targeted-distribution

[32] Soergel A. Nursing homes are getting billions in COVID aid-where is it going? AARP. 2020.

[33] Kingsley DE Harrington C. Financial and quality metrics of A large, publicly traded U.S. nursing home chain in the age of Covid-19. Int J Health Serv. 2022;52(2):212-224. doi:10.1177/00207314221077649 35118905

[34] Harrington CA Hailer L Mollot RJ Mukamel DB. Examining California nursing home profitability and related factors before and during the COVID-19 pandemic. J Am Geriatr Soc. 2023;71:2530-2538. 37026588 10.1111/jgs.18356

[35] Pradhan R Weech-Maldonado R Harman JS Laberge A Hyer K. Private equity ownership and nursing home financial performance. Health Care Manage Rev. 2013;38(3):224-233. 22609748 10.1097/HMR.0b013e31825729ab

[36] CMS. Electronic staffing data submission payroll-based journal. 2022. https://www.cms.gov/Medicare/Quality-Initiatives-Patient-Assessment-Instruments/NursingHomeQualityInits/Downloads/PBJ-Policy-Manual-Final-V25-11-19-2018.pdf

[37] CMS. Medicare provider reimbursement report manual. Chapter 41, Skilled nursing facility and skilled nursing facility health care complex cost report. Form CMS-2540-10. Centers for Medicare & Medicaid Services. https://www.cms.gov/regulations-and-guidance/guidance/manuals/paper-based-manuals-items/cms021935

[38] Harrington C Mollot R Braun RT Williams D. United States’ nursing home finances: spending, profitability, and capital structure. Int J Soc Déterm Heal Heal Serv. 2023. doi:10.1177/27551938231221509 PMC10955796 38115716

[39] Weech-Maldonado R Lord J Davlyatov G Ghiasi A Orewa G. High-minority nursing homes disproportionately affected by COVID-19 deaths. Front Public Health. 2021;9:1-8. doi:10.3389/fpubh.2021.606364 PMC8019707 33829006

[bibr40-00469580241240698] Prusynski RA Leland NE Frogner BK Leibbrand C Mroz TM. Therapy staffing in skilled nursing facilities declined after implementation of the patient-driven payment model. J Am Med Dir Assoc. 2021;22:2201-2206. 33965404 10.1016/j.jamda.2021.04.005 PMC8478699

[bibr41-00469580241240698] McGarry BE White EM Resnik LJ Rahman M Grabowski DC. Medicare’s new patient driven payment model resulted in reductions in therapy staffing in skilled nursing facilities: study examines the effect of Medicare’s patient driven payment model on therapy and nursing staff hours at skilled nursing facilities. Health Aff. 2021;40(3):392-399. 10.1377/hlthaff.2020.00824 33646861

[bibr42-00469580241240698] Lord J Davlyatov G Thomas KS Hyer K Weech-Maldonado R. The role of assisted living capacity on nursing home financial performance. Inquiry; The Journal of Health Care Organization, Provision, and Financing. 2018;55:1-9. 10.1177/0046958018793285 PMC6109846 30141704

[bibr43-00469580241240698] Weech-Maldonado R Laberge A Pradhan R Johnson C Hyer K. Nursing home financial performance: the role of ownership and chain affiliation. Health Care Manage Rev. 2012;37(3):235-245. 22261667 10.1097/HMR.0b013e31823dfe13

[44] Weech-Maldonado R Pradhan R Dayama N Lord J Gupta S. Nursing home quality and financial performance: is there a business case for quality? Inquiry: The Journal of Health Care Organization, Provision, and Financing. 2019;56:1-8. 10.1177/0046958018825191 PMC6376502 30739511

[45] Li Y Cai X Mao Y Cheng Z Temkin-Greener H. Trends in racial and ethnic disparities in coronavirus disease 2019 (COVID-19) outcomes among nursing home residents. Infect Control Hosp Epidemiol. 2021;1-7. 34130766 10.1017/ice.2021.246 PMC8220019

[bibr46-00469580241240698] Li Y Temkin-Greener H Shan G Cai X. COVID-19 infections and deaths among Connecticut nursing home residents: facility correlates. J Am Geriatr Soc. 2020;68(9):1899-1906. 32557542 10.1111/jgs.16689 PMC7323378

[47] McGarry BE Grabowski DC Barnett ML. Severe staffing and personal protective equipment shortages faced by nursing homes during the COVID-19 pandemic. Health Aff. 2020;39(10):1812-1821. 10.1377/hlthaff.2020.01269 PMC7598889 32816600

[48] Allison PD. Fixed Effects Regression Methods for Longitudinal Data Using SAS. SAS Publishing. 2005.

[49] Gibson DM Greene J. State actions and shortages of personal protective equipment and staff in US nursing homes. J Am Geriatr Soc. 2020;68(12):2721-2726. 33022757 10.1111/jgs.16883 PMC7675486

[50] CMS. Trump administration unveils enhanced enforcement actions based on nursing home COVID-19 data and inspection results. CMS News, 2020.

[51] AHCA. Financial Struggle of Nursing Homes Puts Medicaid Reimbursement Rates Back in the Spotlight. American Health Care Association; 2020. https://www.ahcancal.org/News-and-Communications/Press-Releases/Pages/Financial-Struggle-of-Nursing-Homes-Puts-Medicaid-Reimbursement-Rates-Back-in-the-Spotlight.aspx

[52] Xu H Intrator O Bowblis JR . Shortages of staff in nursing homes during the COVID-19 pandemic: what are the driving factors? J Am Med Dir Assoc. 2020;21(10):1371-1377. 32981663 10.1016/j.jamda.2020.08.002 PMC7418696

