# Supplementary material for: COVID-19 Pandemic Impact on Nursing Homes Financial Performance
Source: Inquiry. 2024 Mar 21;61:00469580241240698. doi: 10.1177/00469580241240698 (PMC10958812; doi:10.1177/00469580241240698)
Supplement: sj-docx-1-inq-10.1177_00469580241240698 – Supplemental material for COVID-19 Pandemic Impact on Nursing Homes Financial Performance [file sj-docx-1-inq-10.1177_00469580241240698.docx]

**Appendix 1:** *Interaction of COVID-19 with Percentage Medicare*

| **Interaction Variable** | **Operating Revenue  per Resident Day** | **Operating Cost  per Resident Day** | **Operating  Margin** |
| --- | --- | --- | --- |
| Pre COVID-19 -(< 25% Medicare) | 280.530 | 243.001 | 6.067 |
| Pre COVID-19 -(> 25% Medicare) | 378.450 | 321.063 | 9.470 |
| Post COVID-19 -(< 25% Medicare) | 290.795 | 269.481 | 1.557 |
| Post COVID-19-(> 25% Medicare) | 379.824 | 347.806 | 7.021 |
| **Change for (< 25% Medicare)** | **10.265** | **26.480** | **-4.510** |
| **Change for (> 25% Medicare)** | **1.374** | **26.743** | **-2.449** |
